# The Role of Gut Microbiota in Mice With Bile Duct Ligation-Evoked Cholestatic Liver Disease-Related Cognitive Dysfunction

**DOI:** 10.3389/fmicb.2022.909461

**Published:** 2022-05-10

**Authors:** Bowen Yang, Tianning Sun, Yingle Chen, Hongbing Xiang, Jun Xiong, Shiting Bao

**Affiliations:** ^1^Department of Hepatobiliary Surgery, Affiliated Hospital of Guangdong Medical University, Zhanjiang, China; ^2^Department of Anesthesiology and Pain Medicine, Tongji Hospital, Tongji Medical College, Huazhong University of Science and Technology, Wuhan, China; ^3^Department of Anesthesiology, The First Affiliated Quanzhou Hospital of Fujian Medical University, Quanzhou, China; ^4^Hepatobiliary Surgery Center, Union Hospital, Tongji Medical College, Huazhong University of Science and Technology, Wuhan, China

**Keywords:** cholestatic liver disease, bile duct ligation, cognitive dysfunction, gut microbiota, hepatic encephalopathy

## Abstract

The pathogenesis of Hepatic Encephalopathy (HE) is complex and multifactorial. The development of metagenomics sequencing technology led to show the significant role of gut microbiota in the pathogenesis of cognitive dysfunction, which paved the way for further research in this field. However, it is unknown whether gut microbiota plays a role in bile duct ligation (BDL)-evoked cholestatic liver disease-related cognitive dysfunction. The aim of this investigation is to assess BDL mice induced cognitive dysfunction and meanwhile to delineate the alteration of gut microbiota in cognitive dysfunction mice, which may underline the role of gut microbiota in BDL mice induced cognitive dysfunction. Our study was carried out in male C57BL/6 J mice with bile duct ligation. The liver functions were assessed *via* different biochemical markers [alanine aminotransferase (ALT), aspartate aminotransferase (AST), alkaline phosphatase (ALP), total bilirubin (TBIL), and total bile acid (TBA)] and a histopathological examination of the liver tissue. We used the novel object recognition test (NORT) to assess cognitive dysfunction. And BDL mice were divided into BDL with cognitive dysfunction (BDL-CD) or BDL without cognitive dysfunction (BDL-NCD groups) by the result of hierarchical cluster analysis of NORT. Then, 16S ribosomal RNA (rRNA) gene sequencing was used to compare the gut bacterial composition between BDL-CD and BDL-NCD groups. According to our results, we concluded that bile duct ligation can significantly change the gut microbiota composition, and *Bacteroides fragilis*, *Bacteroides ovatus V975*, and *Bacteroides thetaiotaomicron* play a vital role in BDL-evoked cholestatic liver disease-related cognitive dysfunction.

## Introduction

Hepatic encephalopathy (HE) is mainly manifested by impaired neurological function, including cognitive impairment, abnormal behavior, and coma. (Lockwood et al., 1991; Ferenci et al., 2002; Stewart and Smith, 2007; Vilstrup et al., 2014) Due to the complex pathogenesis of HE, limited progress in the study of HE has been achieved. It is considered that the neurotoxin ammonia and the presence of systemic and neurological inflammation drive the pathogenesis of HE (Sherlock et al., 1954; Shawcross et al., 2011; Gow, 2017; Ninan and Feldman, 2017; Irvine et al., 2019). Rather than independent actions, accumulating evidence points toward a synergic effect among participation factors that contribute to the development and severity of HE (Shawcross et al., 2004; Wright et al., 2007; Ochoa-Sanchez and Rose, 2018).

Gut microbiota has been shown to play a vital role in maintaining the physiological function of the body (Ouwehand et al., 2002; O'Toole and Jeffery, 2015; Sender et al., 2016). With the development in microbiome analysis technology and the many in-depth studies of liver diseases, the role of gut microbiota in liver diseases has attracted more attention. Over the past 20 years, researchers have gradually realized that gut microbiota is involved in the pathogenesis of HE. Liu et al. (2004) first demonstrated a large number of disturbances in the gut microbiota of patients with minimal hepatic encephalopathy (MHE) cirrhosis, with significant overgrowth of potentially pathogenic *Escherichia coli* and *staphylococci* in the stool. Through pyrosequencing of 16S ribosomal RNA, Chen et al. (2011) confirmed that the fecal microbial community of patients with cirrhosis was different from that of healthy people. It is well known that lactulose modulates intestinal flora and is therefore the first-line traditional treatment in chronic liver disease-related HE to reduce urease-producing bacteria. This further demonstrates the role of intestinal flora in HE.

Several landmark papers were published on the relationship between intestinal flora and HE. In 2012, Bajaj et al. demonstrated that patients with cirrhosis, especially when complicated with HE, were associated with significant alterations in the stool microbiome compared with healthy individuals. Specific bacterial families (*Alcaligeneceae, Porphyromonadaceae, and Enterobacteriaceae*) were strongly associated with cognition in HE (Bajaj et al., 2012). In 2015, Bajaj found cirrhotics to have a significantly lower relative abundance of autochthonous taxa compared with healthy controls, and this was further reduced in prior-HE vs. no-HE patients. The cirrhosis dysbiosis ratio (*Lachnospiraceae + Ruminococcaceae + Clostridiales Incerate Sedis XIV + VeilloneIIIaceae/Enterobactericeae + Bacteroidaceae*) was significantly lower (indicating dysbiosis) in cirrhotics compared with controls and significantly worse in prior-HE (Bajaj et al., 2015). Clinical and preclinical studies have directly demonstrated the important role of intestinal flora in brain changes and the development of cognitive dysfunction in HE patients (Ahluwalia et al., 2014; Kang et al., 2016). In a study on 147 patients with cirrhosis and 40 healthy controls, Ahluwalia et al. (2016) indicated that compared with the group of healthy controls and the group with no-HE cirrhosis, patients with HE had greater cognitive impairment and higher relative abundance of local taxa as well as high abundance of *Staphylococci Enterococci, Porphyromonas*, and *Lactobacillaceae*.

The most commonly used model to study HE is the hepatotoxin-induced acute liver failure model. In this study, BDL mice were used to induce cholestatic liver disease to explore the cognitive dysfunction of mice in chronic liver disease. The mice were divided into two groups: cognitive dysfunction (CD)-like phenotype and non-cognitive dysfunction (NCD)-like phenotype according to the results of hierarchical cluster analysis of novel object recognition test (NORT). Meanwhile, 16S ribosomal RNA (rRNA) gene sequencing was used to compare the gut bacterial composition between CD and NCD phenotypes in mice with cholestatic liver disease.

## Materials and Methods

### Animals

Male C57BL/6 J mice, 6–8 weeks of age, were purchased from the Experimental Animal Research Center of Hubei Province (Hubei, China). For acclimatization, they were kept at 23 ± 1°C room temperature under a relative humidity of 55 ± 5% and 12/12-h light/dark cycle for 1 week. The study protocol was approved by the Animal Care and Use Committee of Tongji Hospital, Tongji Medical College (no. TJ0803).

### Experimental Design

The mice were randomly assigned to two groups (1): Control group (Control, *n* = 8) (2); BDL group. Then, BDL mice were divided into cognitive dysfunction like phenotype group (BDL-CD, *n* = 7) and non-cognitive dysfunction like phenotype group (BDL-NCD, *n* = 10) according to the results of hierarchical cluster analysis of NORT.

### Surgery for Bile Duct Ligation

In the control group, the bile duct was separated, and the abdominal cavity was immediately closed. The model of bile duct ligation was prepared following literature (Wang et al., 2017), and the specific process is described as follows: (1) The mice were anesthetized through intraperitoneal injection of 1% sodium pentobarbital (0.01 ml/g). (2) The abdominal fur of the mice was removed with an electric fur shaver, and then, they were placed on a 37°C thermostatic heating plate. The abdominal skin was sterilized with iodine (2–3 times). (3) A 2-cm cut was made in the abdomen, a holding suture was inserted in the sternum, and a retractor was used to enlarge the peritoneal cavity. (4) The liver was lifted with a moistened and warmed cotton swab, such that the ventral side of it stuck to the diaphragm and the hilum was clearly visible. Then, the bile duct was exposed by caudal movement of the gut. (5) The bile duct was separated by placing a 5–0 silk suture around it and securing the suture with two surgical knots. A second ligation was added in the same manner but did not dissect the bile duct in between. (6) The sternum was lowered, and the retractor was removed. Then, a 0.9% NaCl solution (0.3–0.5 ml) was applied to the peritoneal cavity, and the abdominal organs were placed in their physiological positions. (7) The peritoneum and skin were closed using simple continuous suture with 5–0 silk suture, and the operation area was sterilized with iodine. The mice were then placed in a warm environment to resuscitate.

### Behavior Test

The open field test (OFT), NORT, and Y-maze test (Y-Maze) were conducted to assess the cognitive impairment. Behavioral data were automatically analyzed using a smart video tracking system.

#### Open Field Test

The mice were placed at the center of a gray polyethylene box with an open field chamber (L × W × H: 40 cm × 40 cm × 40 cm). They moved freely in dim light for 5 min, and the total distance traveled, motion time and speed were analyzed.

#### Novel Object Recognition Test

The NORT test was performed as follows: In an open field, two identical objects were placed at two corners, 6 cm away from each border, as previously described. At the first stage, the animal was allowed to perform free exploration for 5 min, and the exploration time around each object was recorded. At the second stage, a similar experiment was performed, except that one of the two objects was replaced by a novel object that had the same size but a different appearance. The exploration time around the novel object (NT) and familiar object (FT) were recorded. Recognition Index = (NT−FT)/(NT + FT). After each experiment, the apparatus was wiped by 75% ethanol to eliminate odor after each experiment.

#### Y-Maze

The Y-maze device is made of gray polyethylene and consists of three arms each at an Angle of 120° (L × W × H: 30 cm × 8 cm × 15 cm). These three arms were randomly assigned as follows: the initial arm for the initial animal to be placed and start exploring (always open), the new arm (blocked on the first trial but opened on the second one), and the other arm (always open). At the first stage, the mice were placed in the starting arm (while the new arm was blocked), allowed to explore for 10 min, and then returned to the cage. After 2 h, the second stage was performed, in which the mice were placed in the same starting arm (but with the new one open) and allowed to explore the maze for 5 min. We recorded the time spent in each arm, number of mice entering the new arm, the total number, and the movement distance of the mice for analysis.

### Tissue Sample Collection

#### Fecal Samples

The mice were placed in a clean cage with sterile paper on the bottom. Fecal samples were collected immediately after defecating in a sterilized centrifuge tube. The filter paper was changed after each mouse as our previously study (Li et al., 2021). Fecal samples were stored in a − 80°C freezer. Then, 16S rRNA gene sequencing of fecal samples was performed at OEBiotech Co., Ltd. (Shanghai, China). DNA extraction was performed using the DNA Extraction Kit (Tiangen Biotechnology Co., Ltd., Beijing, China.). *V*3–*V*4 (or *V*4–*V*5) variable regions of 16S rRNA genes were amplified using universal primers 343 F and 798 R. Finally, PCR products were purified for further sequencing. Clean reads were subjected to primer sequences removal and clustering to generate operational taxonomic units (OTUs) using Vsearch software with 97% similarity cutoff. All representative reads were annotated and blasted against Silva database Version 123 (or Greengens) using RDP classifier (confidence threshold was 70%). All representative reads were annotated and blasted against Unite database (ITSs rDNA) using blast.

#### Serum and Liver Samples

The mice were anesthetized *via* intraperitoneal injection of 1% sodium pentobarbital (0.01 ml/g), and the eyeball was removed to collect blood samples. After centrifugation, serum was stored at a −80°C freezer to later perform biochemical analysis. For each mouse of each group, the same lobe of liver was carefully dissected, washed with normal saline, and fixed in 5% neutral formaldehyde solution. Biochemical and histological analyses were performed by Wuhan Servicebio Technology Co., Ltd.

### Statistical Analysis

All quantification data were expressed as means ± SEM, with error bars representing SEM. Statistical analyses were performed using the SPSS software version 25.0 (SPSS Inc., Armonk, New York, United States) and GraphPad Prism software (GraphPad Software, Inc.). Z scores were standardized in the hierarchical cluster analysis. Then, using the Ward method and square Euclidean distance as a distance measurement, NORT results were hierarchically clustered, and the mice were divided into two groups: BDL-CD and BDL-NCD. Behavioral tests were analyzed using one-way or two-way ANOVA, followed by *post-hoc* Bonferroni’s test. *p* < 0.05 was considered to be statistically significant.

## Results

### Biochemical and Histological Changes in the Liver in the BDL Group

**Figure 1:**
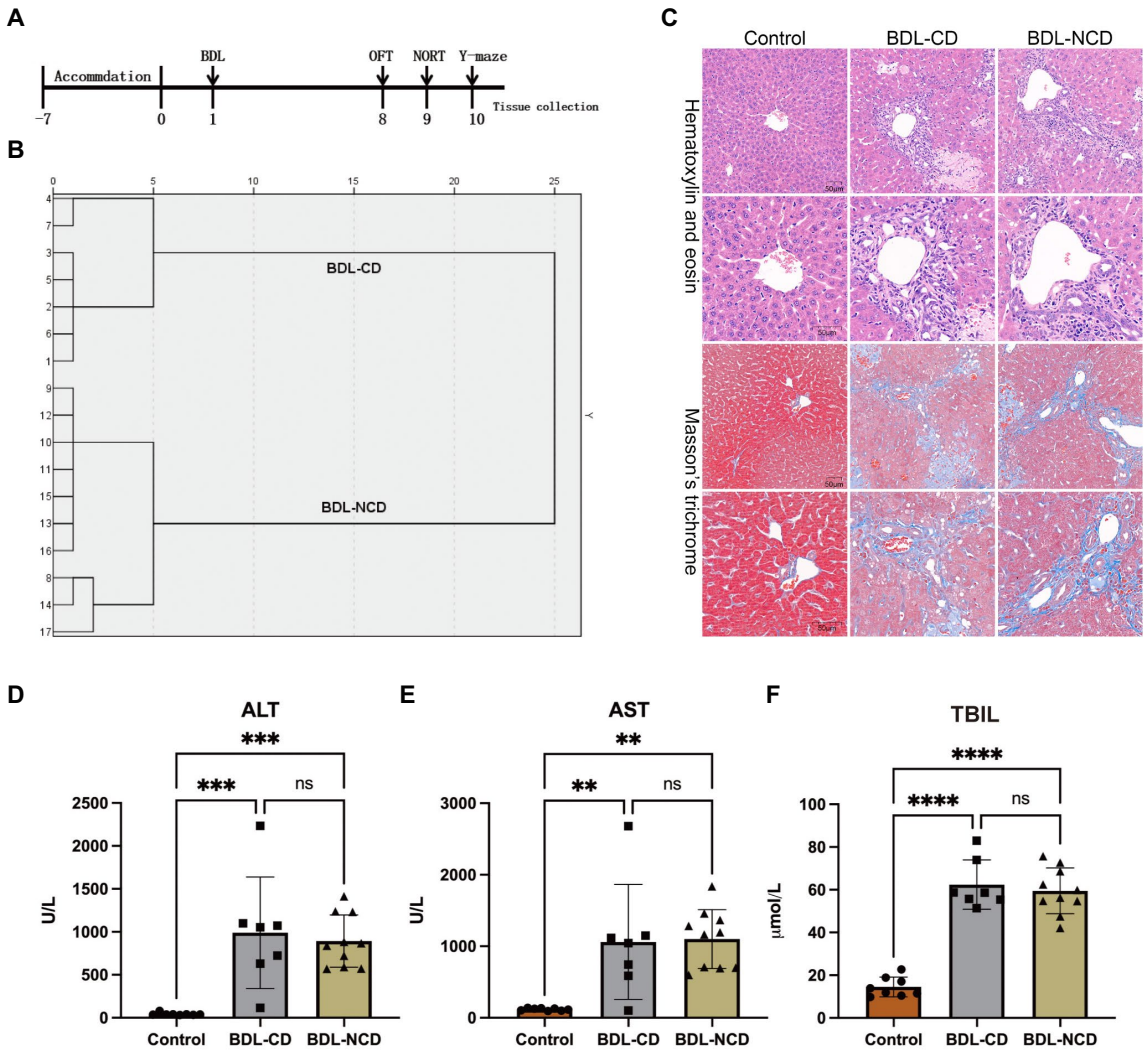
Biochemical and histological changes in the liver in the BDL group. **(A)** Timeline of the BDL model and behavior tests. Ligation of the bile duct in the mice was performed after 7 days of accommodation. The OFT, NORT, and Y-maze tests were carried out on days 8, 9, and 10, respectively. Tissue was collected after the behavior tests. **(B)** Dendrogram of the hierarchical clustering analysis. A total of 17 BDL mice were divided into cognitive dysfunction (BDL-CD, *n* = 7) and no cognitive dysfunction (BDL-NCD, *n* = 10) groups according to the results of hierarchical clustering analysis on NORT results. Before tissue was collected, one mouse died in the BDL-NCD group. **(C)** Hematoxylin & eosin and Masson’s trichrome stained sections of the liver. **(D)** Serum ALT levels. **(E)** Serum AST levels. **(F)** Serum TBIL levels. ALT, alanine aminotransferase; AST, aspartate aminotransferase; and TBIL, total bilirubin. Data are presented as the mean ± SEM. ^**^p < 0.01; ^***^p < 0.001; ^****^p < 0.0001; and ns, not significant.

A timeline of the BDL model and behavior tests are shown in Figure 1A. The results of hierarchical cluster analysis of NORT are shown in Figure 1B. Compared with the control group (Control group), HE and Masson staining indicated apparent liver fibrosis (Figure 1C). Besides, serum ALT, AST, and TBIL levels showed a significant increase (Figures 1D–F).

### Cholestatic Liver Disease-Related Cognitive Dysfunction in Mice

**Figure 2:**
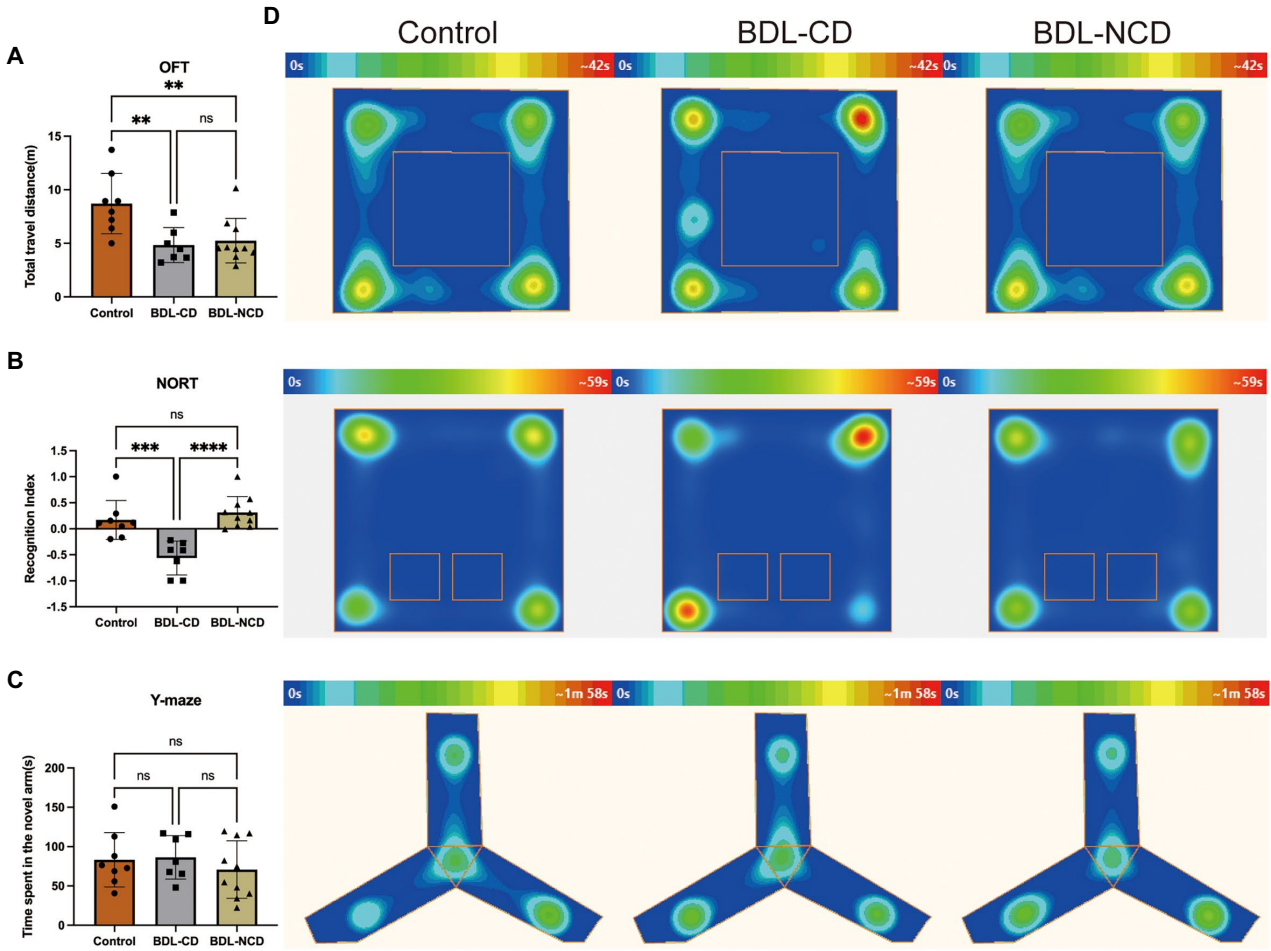
Cholestatic liver disease-related cognitive dysfunction in mice. **(A)** Total travel distance of OFT at day 8. **(B)** Recognition index of NORT at day 9. **(C)** Y-maze test at day 10. **(D)** Heat map of the group center in the OFT, NORT, and Y-maze tests. BDL, Bile Duct Ligation; OFT, Open Field Test; and NORT, Novel Object Recognize Test. Data are presented as the mean ± SEM. ^**^p < 0.01; ^***^p < 0.001; ^****^p < 0.0001; and ns, not significant.

Compared with the Control group, the total travel distance of the BDL-CD and BDL-NCD groups showed a significant decrease, but there was no difference between the two groups (Figure 2A). The Recognition Index of the BDL-CD group was significantly decreased, but there was no significant change between the control and BDL-NCD groups (Figure 2B). Meanwhile, according to the results of the Y-maze test, we did not find a significant change among the three groups (Figure 2C). The heat map of the behavior tests is shown in Figure 2D.

### Community Structure and Diversity Alteration in Gut Microbiota among the Three Groups

**Figure 3:**
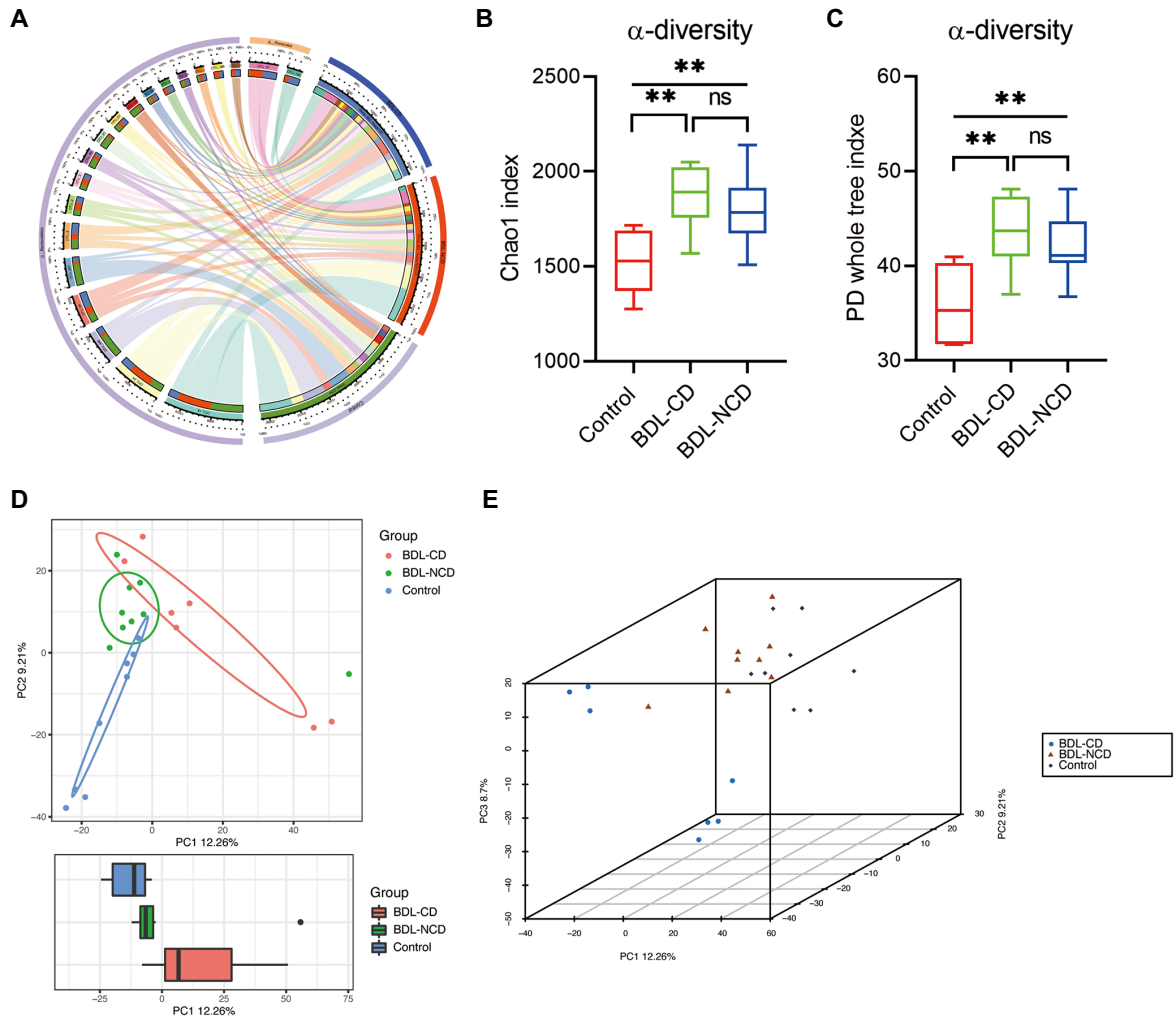
Community structure and diversity alteration in gut microbiota among the three groups. **(A)** Circos plot showing the different community structures among the three groups. **(B)** α-diversity Chao1 index [*F*_(2,21)_ = 8.910, *p* = 0.0016]. **(C)** α-diversity PD whole tree index [*F*_(2,21)_ = 9.043, *p* = 0.0015]. **(D)** Boxplot of PCA. **(E)** 3D plot of PCA. Data are presented as the mean ± SEM. ^**^p < 0.01; ns, not significant.

In order to explore the community structure and diversity of gut microbiota within the Control, BDL-CD and BDL-NCD groups, we drew the Circos plot and calculated the alpha and beta diversity. The Circos plot showed a largely different community structure among the three groups, such that *Bacteroidetes* and *Firmicutes* were the predominant bacteria in mice, and a significant increase in *Firmicutes* was observed in BDL mice (Figure 3A). For alpha diversity, the Chao1 and PD whole tree index indicated a significant increase in BDL mice, but there was no significant change between the BDL-CD and BDL-NCD groups (Figures 3B,C). Beta diversity of PCA showed the gut microbiota to be far apart from each other (Figures 3D,E).

### Differentially Abundant Bacterial Taxa of the Control and BDL Mice

**Figure 4:**
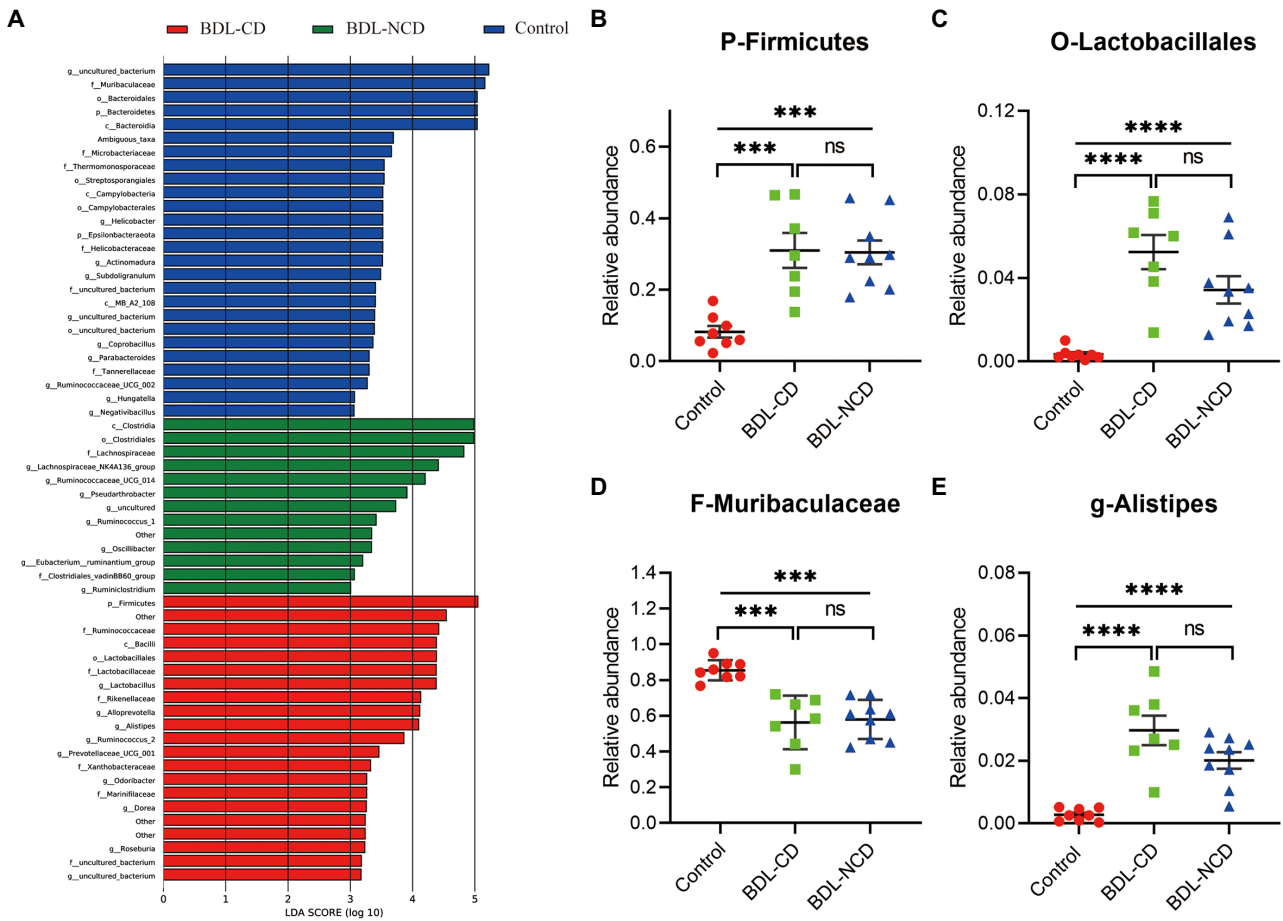
Differentially abundant bacterial taxa between the control and BDL mice. **(A)** LEfSe analysis among the three groups. LefSe, Linear discriminant analysis Effect Size. **(B)** Relative abundance of the phylum *Firmicutes*. **(C)** Relative abundance of the order *Lactobacillales*. **(D)** Relative abundance of the family *Muribaculaceae*. **(E)** Relative abundance of the genus *Alistipes*. Data are presented as the mean ± SEM. ^***^p < 0.001; ^****^p < 0.0001; and ns, not significant.

To identify the types of bacterial taxa that varied among the three groups, we performed the LEfSe (Linear discriminant analysis Effect Size) analysis. LEfSe (Figure 4A) identified 60 discriminative features with relative abundances that significantly varied among the three groups. Next, we performed statistical analysis at all levels of the phyla, class, order, family, genera, and species. The analysis showed that 96 bacteria in the fecal samples were significantly altered among the three groups (phylum:4, class:7, order:9, family:14, genus:26, and species:36). We selected the top 10 different species abundances to obtain the abundance of the dominant different species within groups and comparisons. Compared with the control group, the relative abundance levels of *Firmicutes*, *Clostridia*, *Bacilli*, *Clostridiales*, *Lactobacillales*, *Lactobacillaceae*, *Lachnospiraceae*, *Rikenellaceae*, *Lactobacillus*, *Alistipes*, and *Lactobacillus murinus* were significantly increased in BDL mice (Figures 4B,C,E; Supplementary Figure 1). The relative abundance levels of *Bacteroidetes*, *Bacteroidia*, MB-A2-108, *Erysipelotrichia*, *Bacteroidales*, *Erysipelotrichales*, *Muribaculaceae*, *Tannerellaceae*, *Erysipelotrichaceae*, *Parabacteroides*, and GCA-900066225 were significantly decreased in BDL mice (Figure 4D; Supplementary Figure 2). No statistically significant difference was observed between the BDL-CD and BDL-NCD groups. Compared with the other two groups, the relative abundance levels of *Negativicutes*, *Sphingomonadales*, *Selenomonadales Sphingomonadaceae*, *Bacteroides ovatus* V975, *Bacteroides thetaiotaomicron*, and *Bacteroides_ ragilis* were significantly increased in the BDL-CD group (Supplementary Figure 3).

KEGG pathway prediction heat map indicated that the higher abundance difference in the BDL-CD group is evenly related to Cellular processes and signaling, Cell communication, Sensory system, Carbohydrate metabolism, Lipid metabolism, Genetic information processing, Poorly characterized, Enzyme families, and Metabolism (Supplementary Figure 4).

### The Relationship Between Dysbiosis Markers and Cognitive Dysfunction

**Figure 5:**
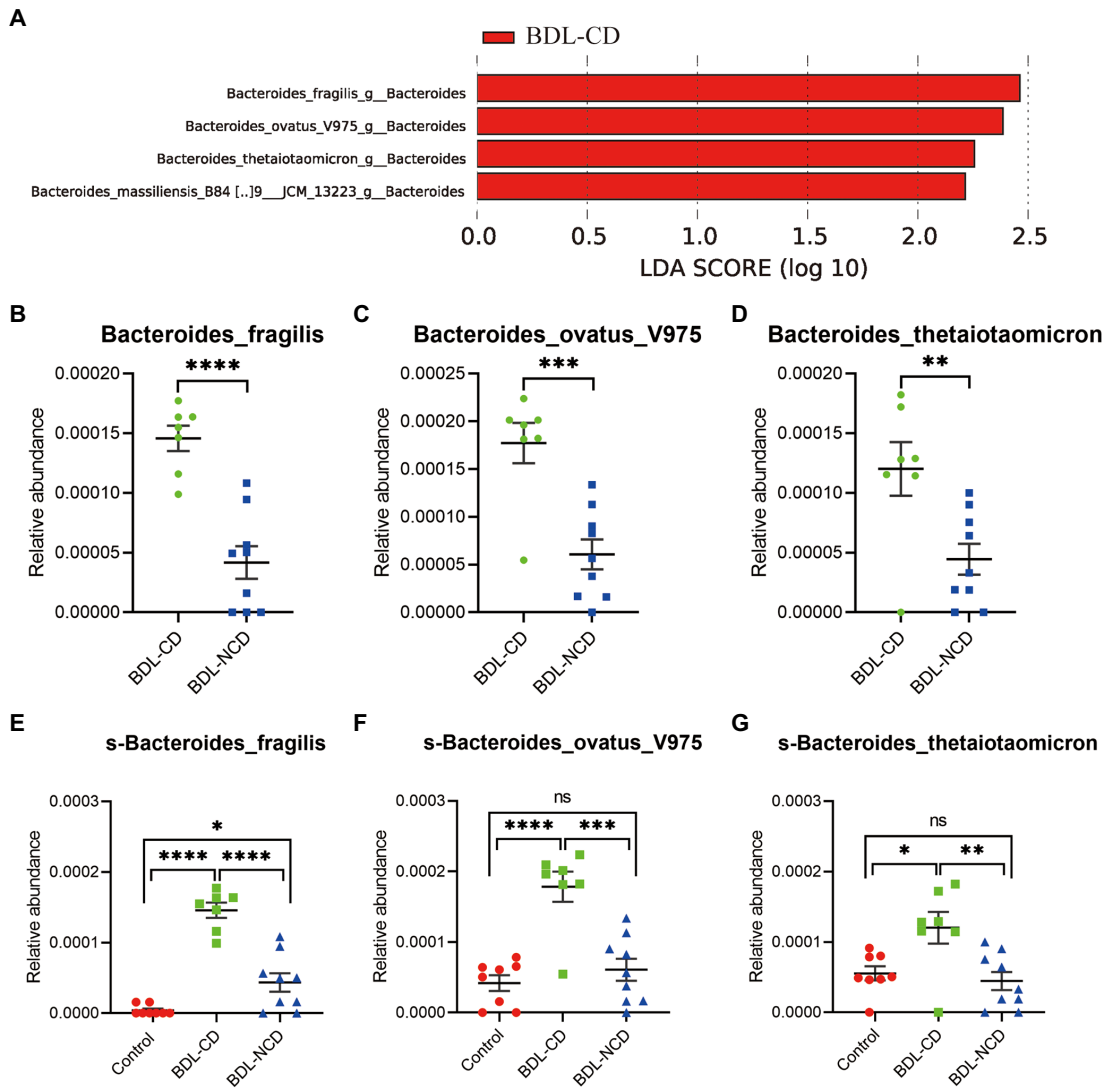
The relationship between dysbiosis markers and cognitive dysfunction. **(A)** Linear discriminant analysis effect size between the BDL-CD and BDL-NCD groups. **(B–G)** Relative abundance of gut microbiota at the species level. Data are presented as the mean ± SEM. ^*^p < 0.05; ^**^p < 0.01; ^***^p < 0.001; ^****^p < 0.0001; and ns, not significant.

In this further analysis, we focus on the relationship between dysbiosis markers of microbiota and cognitive dysfunction among the BDL-CD and BDL-NCD groups. We employed LEfSe analysis at all levels of bacteria between the two groups. We observed four markers at the species level (Figure 5A), but only three markers showed significant increase in the BDL-CD group (Figures 5B–D), including *Bacteroides fragilis*, *Bacteroides ovatus* V975, and *Bacteroides thetaiotaomicron*. ANOVA analysis and Tukey’s multiple comparison test further assessed the differences in these three markers among the three groups (Figures 5E,F). The results showed that only in the BDL-CD group, the relative abundance of *Bacteroides fragilis*, *Bacteroides ovatus* V975, and *Bacteroides thetaiotaomicron* showed a statistically significant increase, which may play a major role in BDL-evoked cognitive dysfunction.

## Discussion

The novel object recognition test is a valuable measure of cognition, which is mainly used to measure learning and memory according to the differences in the exploration time of novel and familiar objects. This study demonstrated that liver fibrosis was induced, and learning/memory impairment appeared 8 days after bile duct ligation in C57BL/6 mice. Kerfoot et al. (2006) and D'Mello et al. (2009) used male C57BL mice (6–8 weeks of age) to demonstrate that the recruitment of activated monocytes into the brain was observed 10 days after BDL. This implies that inflammatory responses were already triggered in the brain 10 days after BDL. Thus, 8 days after BDL may be suitable to investigate the cognitive dysfunction accompanying liver disease in mice. In our study, learning and memory impairment were observed after BDL, while liver fibrosis and elevation of liver enzymes were also detected.

HE can be divided into type A, type B, and type C. In this work, we used BDL C57BL/6 mice to create a model of type C, which is caused by cirrhosis. We successfully established a BDL-evoked HE model in C57BL/6 mice and found significant biochemical and histopathologic changes in the liver. Our findings were in accordance with previous reports that demonstrated impaired memory in rat models of HE, including the BDL model with impaired working memory through the novel object recognition test (Dhanda et al., 2018).

In recent years, multi-omics techniques have revealed the vital role gut microbiota plays in promoting human health. Hence, exploring the roles and mechanisms of the gut microbiota in cognitive dysfunction in liver diseases represents an emerging field of research. In a recent paper published in Nature, Irish scientists reported that transplanting the intestinal flora of young mice significantly improved the cognitive and behavioral performance of aging mice (Boehme et al., 2021). An important role of intestinal flora in cognitive dysfunction has been further proven by the application of fecal transplantation for the treatment of cognitive-related diseases. Increasing evidence demonstrated the importance of gut microbiota in maintaining normal function of Central Nervous System (CNS). For instance, Ling et al. (2020) found that patients with Alzheimer’s disease (AD) have reduced numbers of butyrate-producing bacteria, such as *Fecealibacterium*, and increased ones of lactate-producing ones, which were both significantly correlated with the host inflammatory cytokines as well as clinical indicators of AD. For liver disease-associated brain dysfunction, the hepato-entero-brain signal axis is expected to provide a new target for diagnosis and treatment and crack the pathogenesis. Therefore, we speculate that cognitive dysfunction induced by ligation of the bile duct in mice may be associated with disturbed gut microbiota.

In our study, we found that the composition of gut microbiota was different between the BDL and Control mice. The relative abundance of *Bacteroidetes* was significantly decreased in BDL mice. On the contrary, there was an obvious increase in *Firmicutes*. It is commonly recognized that a dysfunctional level of the phylum *Firmicutes* and the order *Bacteroidales* is associated with cognitive dysfunction. Hoffman et al. (2017) found that the *Firmicutes/Bacteroidetes* ratio was significantly increased in older mice. Therefore, it seems that the abnormal composition of these microbiota may play a role in BDL-evoked cognitive dysfunction.

LEfSe identified 60 discriminative features with relative abundances that significantly varied among the three groups. A total of 96 bacteria in the fecal samples were significantly altered among the three groups (phylum:4, class:7, order:9, family:14, genus:26, and species:36). The above-mentioned results have only demonstrated a potential association, not causation, between cognitive dysfunction, liver fibrosis, and microbial change. A potential mechanism could be the obvious decrease of bile acid in the intestinal tract, which can modulate the microbiota in the gut (Vlahcevic et al., 1970; Kakiyama et al., 2013; Ridlon et al., 2013). Furthermore, we focused on the relationship between the dysbiosis markers of microbiota and cognitive dysfunction among the BDL-CD and BDL-NCD groups. We employed LEfSe analysis at all levels of bacteria between the two groups and observed four markers at the species level. Only three markers showed a statistically significant increase in the BDL-CD group, including *Bacteroides fragilis*, *Bacteroides ovatus* V975, and *Bacteroides thetaiotaomicron*. ANOVA analysis and Tukey’s multiple comparison test further assessed the differences in these three markers among the three groups. The results showed that the relative abundance of *Bacteroides fragilis*, *Bacteroides ovatus* V975, and *Bacteroides thetaiotaomicron* significantly increased only in the BDL-CD group, which may play a major role in BDL-evoked cognitive dysfunction.

The proportion of *Bacteroidetes* was significantly decreased in BDL mice, but interestingly, we found that the relative abundance of *Bacteroides fragilis*, *Bacteroides ovatus* V975, and *Bacteroides thetaiotaomicron*, which all belong to *Bacteroidetes*, were significantly increased in the BDL-CD mice. A study investigated the effect of lactulose on fecal flora in patients with liver cirrhosis. Ito et al. (1997) demonstrated that lactulose supplementation suppressed the growth of *Bacteroides* spp. (*B. fragilis* group) exerts a beneficial effect on hepatic encephalopathy by decreasing toxic short-chain (iC4-nC6) fatty acid production. In a case report, Matsukawa et al. (2003) found that an alcohol abuser with hepatitis C developed multibacterial sepsis, including *Bacteroides thetaiotaomicron*. As a normal commensal microbe, *Bacteroides fragilis* are usually thought to be advantageous to health due to their abilities of biosynthesis and metabolism (Fathi and Wu, 2016; Lukiw, 2016). However, when enterotoxigenic strains of *Bacteroidetes* species, including *Bacteroides fragilis*, proliferate, and their formidable array of secreted neurotoxins, including the classic neuroinflammatory pattern recognition molecule Lipopolysaccharide (LPS), leak through the normally protective mucosal barriers of the intestinal tract and blood–brain barrier (BBB), they can cause substantial inflammatory pathology, both systemically and within vulnerable CNS compartments (Fathi and Wu, 2016; Lukiw, 2016; Batista et al., 2019; Sheppard et al., 2019). The lipopolysaccharide of *Bacteroides fragilis* is one of the most neurotoxic pro-inflammatory lipoglycans (Sears, 2009; Sheppard et al., 2019). LPS-induced cognitive dysfunction may be related to the result of upregulated neuroinflammatory, attenuated neocortical and hippocampal microglial activation, cytokine and reactive oxidative species generation, oxidative stress damage, and loss of synaptic plasticity-related proteins (Li et al., 2015; Yang and Chiu, 2017; Barton et al., 2019; Batista et al., 2019; Sheppard et al., 2019; Wu et al., 2019). Similar to *Bacteroides fragilis*, *Bacteroides ovatus* V975, and *Bacteroides thetaiotaomicron* are Gram-negative bacteria, which generate nanosized outer membrane vesicles, potentially leading to the same pathological process.

It goes without saying that the maintenance of gut homeostasis relies on an intact gut mucosal barrier and a healthy gut microenvironment. Gut homeostasis disorder is characterized by bacterial overgrowth and translocation. The gut barrier is the first defense barrier against bacteria and their metabolites entering the blood, which can be destroyed due to severe disease. When the gut barrier is destroyed, bacteria and their metabolites enter the circulation more easily. At the same time, gut metabolites and the components of bacteria in the blood, including LPS, Peptidoglycan (PGN), and Bacterial lipoprotein (BLP), are difficult to be broken down in the damaged liver. This leads to systemic inflammation and neuroinflammation, thus promoting the occurrence of HE.

To sum up, we successfully created a model of cholestatic liver disease-related by ligation of the bile duct in mice and used the novel object recognition test to prove the existence of cognitive dysfunction. The results of 16 s RNA sequencing showed that *Bacteroides fragilis*, *Bacteroides ovatus* V975, and *Bacteroides thetaiotaomicron* may play an important role in the cognitive dysfunction of HE. However, further study focusing on the mechanism of neuroinflammation resulted by this flora is needed to confirm the results.

## Data Availability Statement

The original contributions presented in the study are included in the article/Supplementary Material; further inquiries can be directed to the corresponding authors.

## Ethics Statement

The animal study was reviewed and approved by Animal Care and Use Committee of Tongji Hospital, Tongji Medical College (no. TJ0803).

## Author Contributions

All authors listed have made a substantial, direct, and intellectual contribution to the work and approved it for publication.

## Funding

This work was supported by Science and Technology Bureau Project in Technology Bureau Quanzhou City, Fujian Province (no. 2021N074S).

## Conflict of Interest

The authors declare that the research was conducted in the absence of any commercial or financial relationships that could be construed as a potential conflict of interest.

## Publisher’s Note

All claims expressed in this article are solely those of the authors and do not necessarily represent those of their affiliated organizations, or those of the publisher, the editors and the reviewers. Any product that may be evaluated in this article, or claim that may be made by its manufacturer, is not guaranteed or endorsed by the publisher.

## Supplementary Material

Supplementary Figure 1 Relative abundance of gut microbiota. Data are presented as the mean ± SEM. ^**^*p* < 0.01; ^***^*p* < 0.001; ^****^*p* < 0.0001; ns, not significant.

Supplementary Figure 2 Relative abundance of gut microbiota. Data are presented as the mean ± SEM. **p* < 0.05; ***p* < 0.01; ****p* < 0.001; ^****^*p* < 0.0001; ns, not significant.

Supplementary Figure 3 Relative abundance of gut microbiota. Data are presented as the mean ± SEM. **p* < 0.05; ***p* < 0.01; ****p* < 0.001; ^****^*p* < 0.0001; ns, not significant.

Supplementary Figure 4 KEGG pathway prediction heat map based on 16S rRNA.
