# Supplementary figures and images for: The Role of Gut Microbiota in Mice With Bile Duct Ligation-Evoked Cholestatic Liver Disease-Related Cognitive Dysfunction
Source: Front Microbiol. 2022 May 10;13:909461. doi: 10.3389/fmicb.2022.909461 (PMC9127770; doi:10.3389/fmicb.2022.909461)

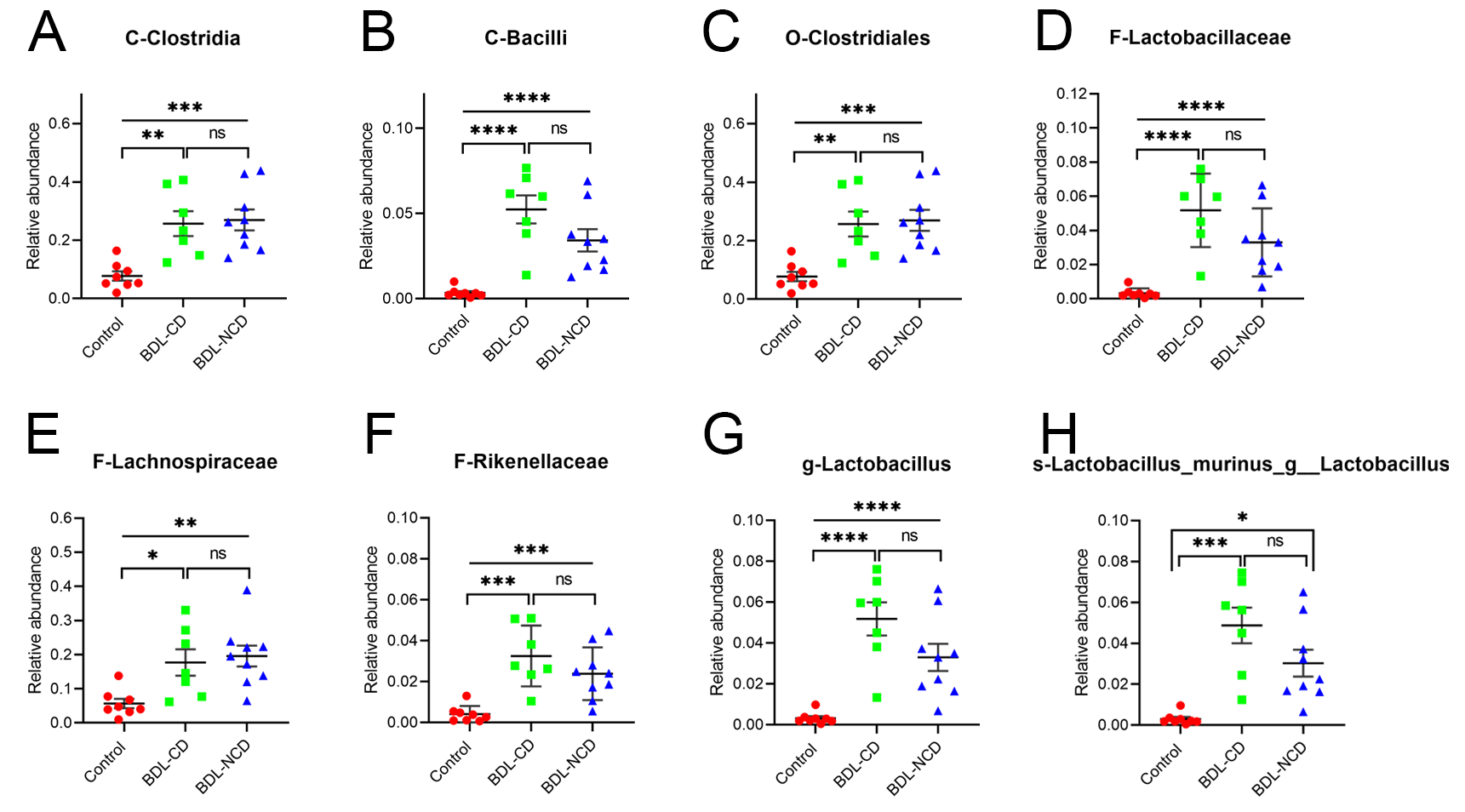

Supplement: Supplementary Figure 1 — Relative abundance of gut microbiota. Data are presented as the mean ± SEM. **p < 0.01; ***p < 0.001; ****p < 0.0001; ns, not significant. [file Image_1.PNG]

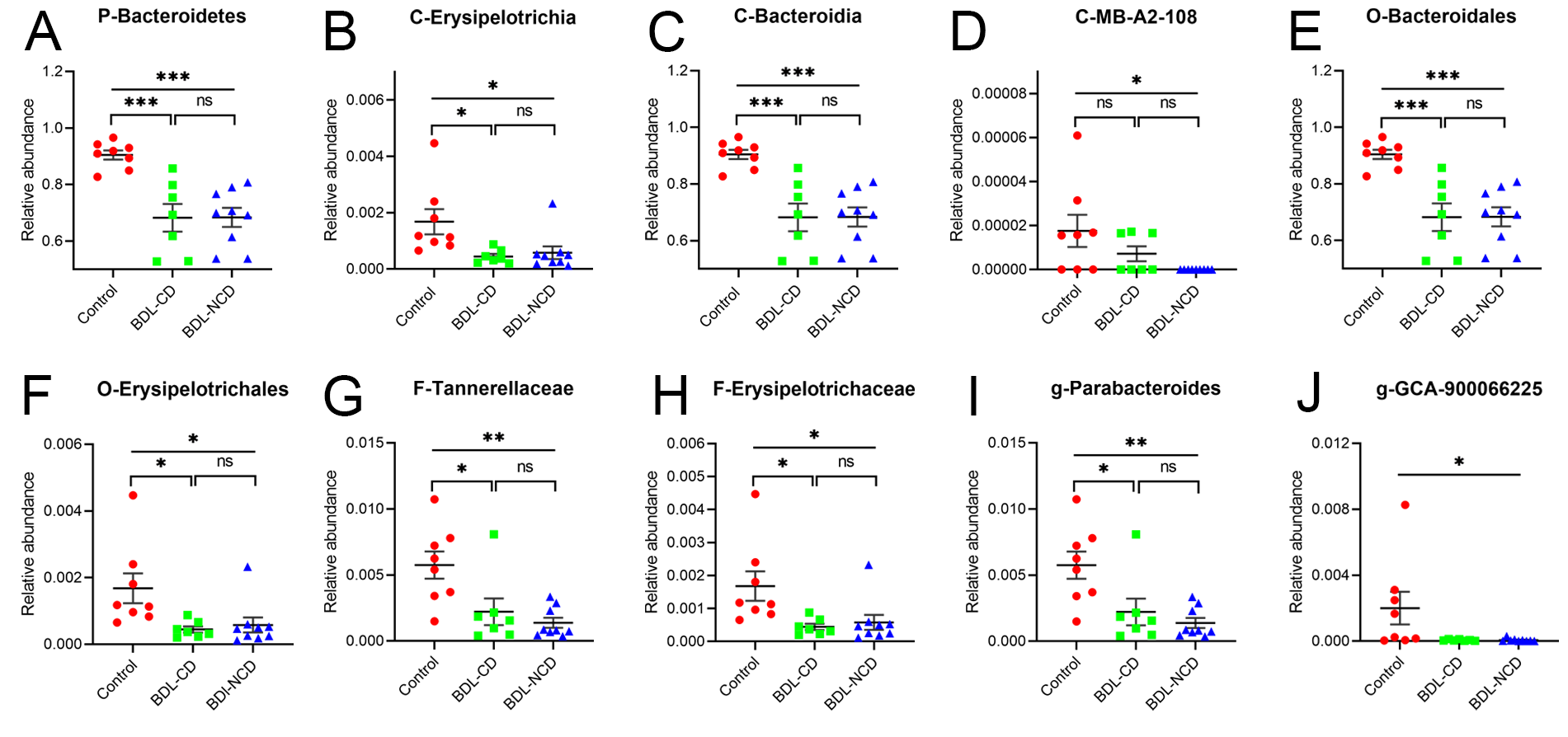

Supplement: Supplementary Figure 2 — Relative abundance of gut microbiota. Data are presented as the mean ± SEM. *p < 0.05; **p < 0.01; ***p < 0.001; ****p < 0.0001; ns, not significant. [file Image_2.PNG]

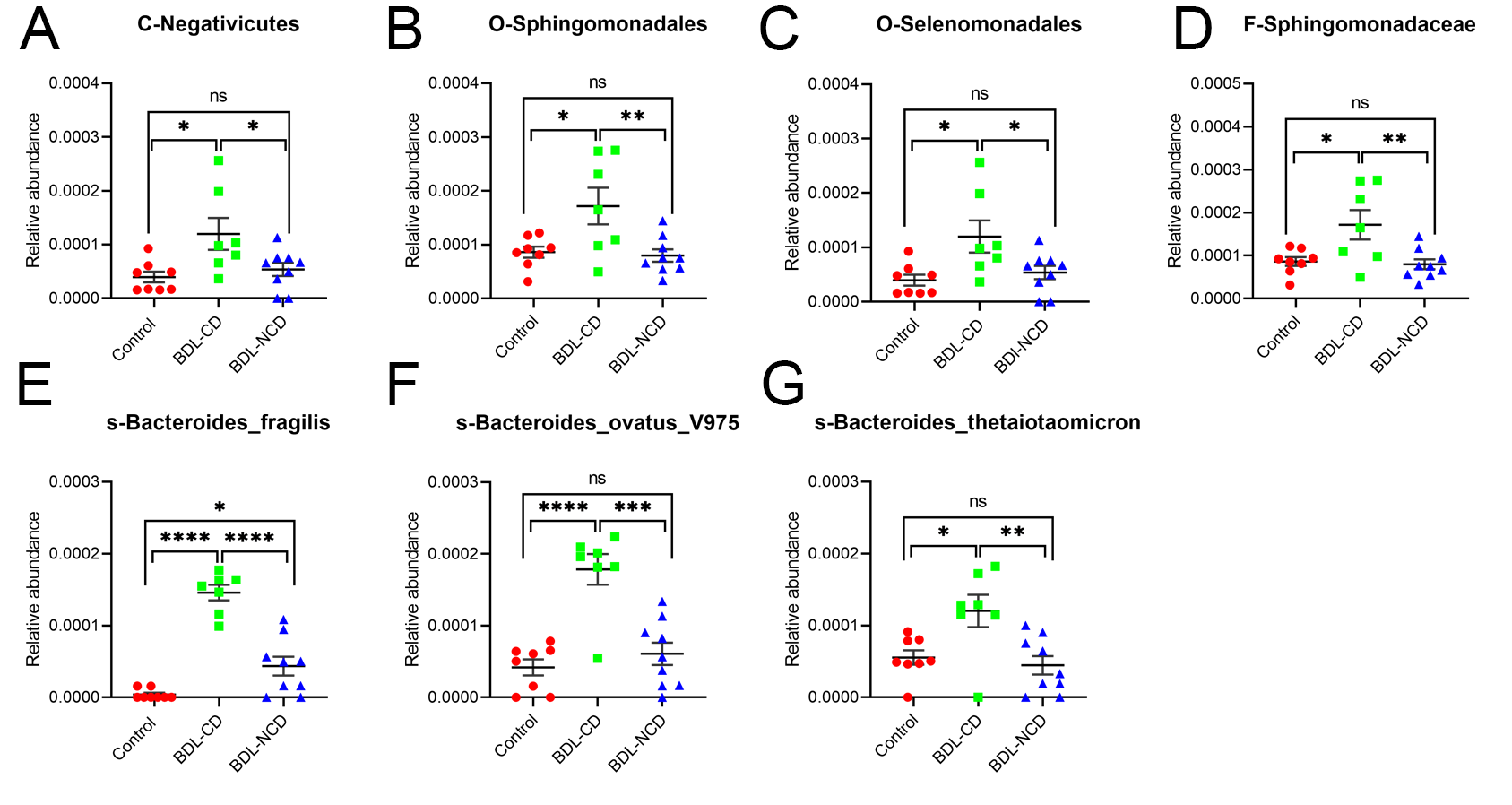

Supplement: Supplementary Figure 3 — Relative abundance of gut microbiota. Data are presented as the mean ± SEM. *p < 0.05; **p < 0.01; ***p < 0.001; ****p < 0.0001; ns, not significant. [file Image_3.PNG]

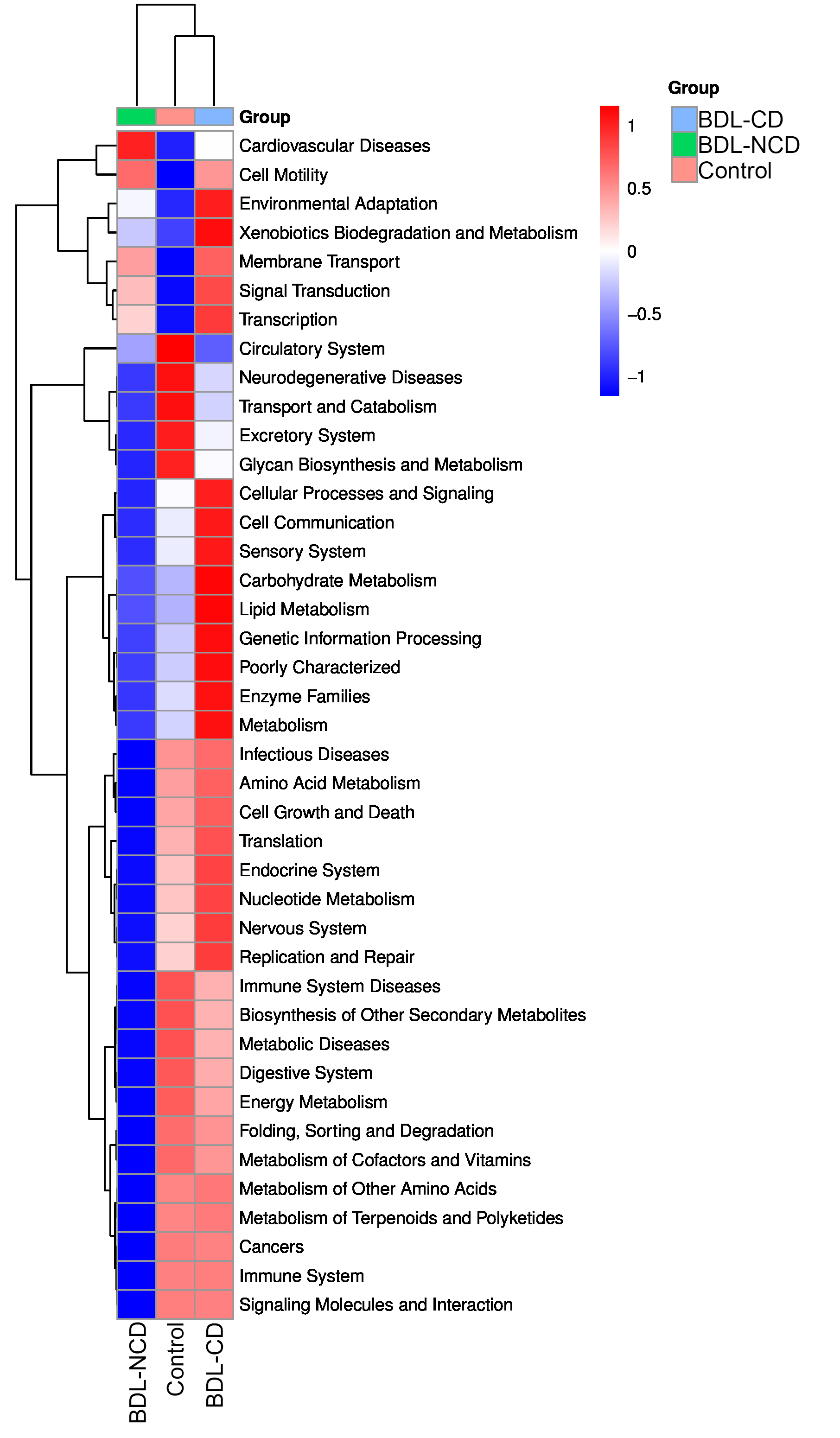

Supplement: Supplementary Figure 4 — KEGG pathway prediction heat map based on 16S rRNA. [file Image_4.TIF]
